# Stochastic non-linear oscillator models of EEG: the Alzheimer's disease case

**DOI:** 10.3389/fncom.2015.00048

**Published:** 2015-04-24

**Authors:** Parham Ghorbanian, Subramanian Ramakrishnan, Hashem Ashrafiuon

**Affiliations:** ^1^Department of Mechanical Engineering, Center for Nonlinear Dynamics and Control, Villanova UniversityVillanova, PA, USA; ^2^Department of Mechanical and Industrial Engineering, University of Minnesota DuluthDuluth, MN, USA

**Keywords:** EEG, Alzheimer's disease, stochastic differential equations, duffing—van der Pol, entropy

## Abstract

In this article, the Electroencephalography (EEG) signal of the human brain is modeled as the output of stochastic non-linear coupled oscillator networks. It is shown that EEG signals recorded under different brain states in healthy as well as Alzheimer's disease (AD) patients may be understood as distinct, statistically significant realizations of the model. EEG signals recorded during resting eyes-open (EO) and eyes-closed (EC) resting conditions in a pilot study with AD patients and age-matched healthy control subjects (CTL) are employed. An optimization scheme is then utilized to match the output of the stochastic Duffing—van der Pol double oscillator network with EEG signals recorded during each condition for AD and CTL subjects by selecting the model physical parameters and noise intensity. The selected signal characteristics are power spectral densities in major brain frequency bands Shannon and sample entropies. These measures allow matching of linear time varying frequency content as well as non-linear signal information content and complexity. The main finding of the work is that statistically significant unique models represent the EC and EO conditions for both CTL and AD subjects. However, it is also shown that the inclusion of sample entropy in the optimization process, to match the complexity of the EEG signal, enhances the stochastic non-linear oscillator model performance.

## 1. Introduction

Quantitative analysis of human brain electroencephalography (EEG) recordings aimed at enhancing our understanding of brain injuries and disorders is currently an important research area. In addition to being useful in diagnosis, such analysis can provide insights into the underlying neurophysiology of the injury or disorder, thereby leading to better treatment and preventive strategies. Alzheimer's disease (AD) is the most common form of dementia and is the subject of intense research. While no known cure exists, certain medications have shown promise in delaying the symptoms (Dauwels et al., 2010) prompting researchers to seek early diagnosis and intervention strategies. In this context, analysis of the EEG is a potential non-invasive tool that may aid early diagnosis of AD. However, the use of EEG signal analysis in order to improve the diagnosis of AD is a complex problem where, despite significant advances, a number of fundamental questions remain open (Elgendi et al., 2011).

Considering now the characteristics of the EEG, since the non-stationary nature of the signal is generally well-recognized (see, for instance Akin, 2002), decomposition using a fast Fourier transform (FFT) with sliding windows and the wavelet transforms have been the most popular techniques employed to capture the spectral properties of EEG (Darvishi and Al-Ani, 2007; Dauwels et al., 2010). However, linear transformation methods fail to address the non-linear characteristics of the EEG signal (Stam, 2005). Therefore, non-linear dynamic approaches have been attempted as well, mostly involving computationally complex time series analysis (Jeong, 2004). Several other aspects of non-linear modeling and analysis in this context have also been studied in the literature (see, for instance, Stam, 2005 for a review). These include frameworks based on a neural mass model (Valdes et al., 1999; Huang et al., 2011), coupled oscillators (Baier et al., 2005; Leistritz et al., 2007), continuum models (Kim et al., 2007), non-linear non-stationary models (Celka and Colditz, 2002; Rankine et al., 2007), random neural networks (Acedo and Morano, 2013), and chaotic phenomena and stability aspects (Rodrigues et al., 2007; Dafilis et al., 2009). Stochastic approaches based on Markov chain Monte Carlo methods (Hettiarachchi et al., 2012) and Markov process amplitude (Wang et al., 2011) that take into account the inherent randomness of the EEG signal have also been reported. In the same vein, limit cycle oscillators (Hernandez et al., 1996; Burke and Paor, 2004) as well as stochastic synchronization (Bressloff and Lai, 2011) and stochastic approximation (Fell et al., 2000; Sun et al., 2008) methods have been considered in EEG modeling. Notably, limit cycle behavior at each of the brain frequency bands appears to provide a more accurate representation of the EEG signal than one based on chaotic phenomena.

Some of the most important features in non-linear dynamic and stochastic approaches are signal information content and complexity as measured using various forms of information entropy. Measures such as Shannon entropy (Shannon, 1948) characterize the information content in a signal and higher entropy corresponds to increased randomness and chaotic behavior (Abasolo et al., 2006). Importantly, one observes that, with respect to the EEG signal, higher information content correlates with better brain function (Shin et al., 2006). Furthermore, it has been reported that variations in information entropic measures may be used to detect functional abnormalities in the brain caused by disorders or injuries (Slobounov et al., 2009). Hence, information content of the EEG signal, characterized by information-entropic measures, may be expected to be important in identifying distinct states of the brain. This is further reinforced by the recent results of McBride and colleagues on the role of information entropic and spectral analysis in the study of the early stages of Alzheimer's disease and mild Traumatic Brain Injury McBride et al. (2013a,b, 2014).

Entropy may also be utilized to measure signal complexity. For instance, embedding entropy provides information about how the EEG signal fluctuates in time by comparing the time series with a delayed version of itself (Abasolo et al., 2006). Moreover, the concept of approximate entropy was introduced as a measure of system complexity (Pincus, 1991) and has been applied to brain wave signals (Quiroga et al., 2001). However, the approximate entropy measure suffers from drawbacks such as bias and inconsistency (Xu et al., 2010). Hence, the notion of sample entropy was introduced (Richman and Moorman, 2000) as an improvement over approximate entropy.

In recent work, the authors proposed a phenomenological model of the EEG signal based on the dynamics of a stochastic, coupled, Duffing- van der Pol oscillator network (Ghorbanian et al., 2015). An optimization scheme was adopted to match model output with actual EEG data obtained from healthy subjects in the two distinct resting eyes-open (EO) and eyes-closed (EC) conditions and it was shown that the actual EEG signals in both cases were distinct realizations of the model with qualitatively different non-linear dynamic characteristics. Moreover, the model output and the actual EEG data were shown to be in good agreement in terms of both the power spectra (frequency content) and Shannon entropy (information content).

In the present effort, we improve the model introduced in Ghorbanian et al. (2015) by matching the sample entropy of the model output and EEG signal to capture its complexity. A global optimization routine is employed in order to match the output of with EEG recordings in terms of power spectrum, Shannon entropy, and sample entropy. The EEG signals were recorded under resting EC and EO conditions in an earlier pilot study of Alzheimer's disease (AD) patients vs. age-matched healthy control (CTL) subjects (Ghorbanian et al., 2013). The model parameters obtained for the oscillators representing EC and EO EEG signals for CTL and AD patients are compared in order to establish statistically significant, distinct models for AD and CTL subjects under each condition. In addition, we present new results from a phase space reconstruction analysis of the model output to match the actual EEG signal. The results indicate that the analytical model effectively captures the frequency spectrum and non-linear characteristics of the EEG signal in terms of complexity and information content. Furthermore, it is shown that the addition of sample entropy significantly enhances the model performance in terms of complexity and non-linear dynamic characteristics, as demonstrated by phase space reconstruction. The results suggest exciting new pathways to develop better tools for distinguishing pathological and normal brain states in AD and perhaps other neurological diseases and disorders.

The rest of the article is set as follows. Details of the EEG recordings, the analytical model, the optimization scheme and the phase space reconstruction technique are provided in Section 2. The results are presented in Section 3 and discussed in Section 4. The articles concludes with comments on further research in Section 5.

## 2. Materials and methods

### 2.1. EEG recording blocks

Twenty six AD patients and healthy age-matched CTL subjects were selected for this study (“A Brain-Computer Interface for Diagnosing Brain Function,” Aspire IRB, Human Subject Protocol Number PDMC-001, approved on October 7, 2010). Of the 26 subjects selected, one withdrew and one did not qualify as AD or CTL. Subjects were asked to relax and wear an EEG recording headset during alternating blocks of EC and EO followed by a variety of cognitive and auditory tasks and a final EC-EO resting period.

The EEG signals were recorded through a single-dry electrode device at position Fp1 (based on a 10–20 electrode placement system) with a Bluetooth enabled telemetric headset. The headset's effective sample rate is 125 Hz. Frequencies below 1 Hz and above 60 Hz (near Nyquist frequency) were filtered out by the device hardware. On comparison of the EEG recordings by the device with those from other widely accepted devices, frequencies within 2–30 Hz were deemed to be very accurate.

The recording device eliminated frequently observed artifacts including line noise. Other artifacts were mainly due to eye- and muscle-movements, which are common at Fp1 location and can be clearly identified by their high amplitudes compared to true EEG signal recordings during resting states. These artifacts were removed using a simple artifact detection that eliminated any part of the signal greater than 4.5σ (standard deviation). The algorithm also reconstructed the nulled samples using FFT interpolation of the trailing and subsequent recorded data (Ghorbanian et al., 2013).

The EEG recordings in this study were obtained from subjects in an AD pilot study with 14 control (CTL) subjects and 10 Alzheimer's Disease (AD) patients presented in our earlier work (Ghorbanian et al., 2013). Recording blocks of 40-s duration (approximately 5000 sample signals) from resting eyes-closed (EC) and eyes-open (EO) conditions were selected. In all, 60 random blocks were selected from the pilot study: 40 blocks from control CTL subjects (20 EC and 20 EO) and 20 blocks from AD subjects (10 EC and 10 EO). Note that, the smaller number of AD patients along with smaller number of AD patient recording sessions that were were not dominated by artifacts resulted in the selection of smaller AD sample size.

### 2.2. EEG features

The time-varying power spectrum in each of the major brain EEG frequency bands was calculated using short time fast Fourier transform (FFT) with sliding window, since a good model must produce signals that can match EEG's frequency content. Specifically, the power spectrum was computed in seven ranges: lower δ (1–2 Hz), upper δ (2–4 Hz), θ (4–8 Hz), α (8–13 Hz), lower β (13–20 Hz), upper β (20–30 Hz), and γ (30–60 Hz). However, lower δ and γ band powers, which happen to have little power, were ignored due to unreliability of the device in those frequency ranges.

Shannon entropy was measured based on a sliding temporal window technique. A temporal window was defined to slide along the signal time representation with a sliding step (interval or bin) to sample a part of the signal. A discrete entropy estimator was applied, in which 10 uniform intervals equally divided the range of the normalized observed signal. Then the probability that the sampled signal belongs to the interval is the ratio between the number of the samples found within each interval and the total number of samples of the signal. The Shannon entropy is then calculated based on these probabilities (Shin et al., 2006), separately for each 40-s EEG recording block (5000 samples).

Sample entropy (SE) is the negative natural logarithm of the conditional probability that two sequences of a time series, similar for *m* points, remain similar at the next point. For given *N* data points from a time series, [*x*(1), *x*(2), …, *x*(*N*)], we calculated SE of each 40-s EEG recording block (5000 samples) by the statistic (Abasolo et al., 2006):

(1) $$\text{SE}(m,r,N)=\left\{-\ln\left[\frac{U^{m+1}(r)}{U^{m}(r)}\right]\right\},$$

where *m* is the run length, *r* is the tolerance window size, and

(2) $$U^{m}(r)=\frac{1}{\left(N-m)(N-m-1\right)}\sum_{i=1}^{N-m}U_{i}.$$

In the above equation, *U*_*i*_ indicates the number of *k*'s (1 ≤ *k* ≤ *N* − *m*) such that the Euclidean distance between *X*_*m*_(*i*) and *X*_*m*_(*k*), *k* ≠ *i*, is less than or equal *r* and *X*_*m*_(*i*) = [*x*(*i*), *x*(*i* + 1), …, *x*(*i* + *m* − 1)].

Generally, large *m* or small *r* values result in number of matches being too small for confident estimation of the conditional probability and vice versa (Lake and Moorman, 2011). In this study, we used *m* = 2 and *r* = 0.25σ based on the consistency of the results and recommended ranges in previous studies (Richman and Moorman, 2000; Xu et al., 2010).

### 2.3. Stochastic coupled non-linear oscillators

We recall that the EEG has been modeled in the literature taking into account characteristics including non-linearity (both chaotic and non-chaotic), non-stationarity, and randomness of the signal (Fell et al., 2000; Rankine et al., 2007; Sun et al., 2008). The EEG has also been studied as the manifestation of underlying limit cycle oscillations at a given frequency and other such periodic solutions (Hernandez et al., 1996; Burke and Paor, 2004). While inspired by the above, the authors were fundamentally motivated to develop models that can better reproduce the significant linear and non-linear characteristics of actual EEG signals. Hence, we proposed a phenomenological model of the EEG based on a coupled system of Duffing—van der Pol oscillators subjected to white noise excitation (Ghorbanian et al., 2015). This particular oscillator was selected because the Duffing non-linearity allows a system with only two oscillators capture the major brain frequency spectra and van der Pol non-linearity provides self-excited limit cycle behavior which have been previously reported for each major brain frequency bands (Burke and Paor, 2004).

**Figure 1:**
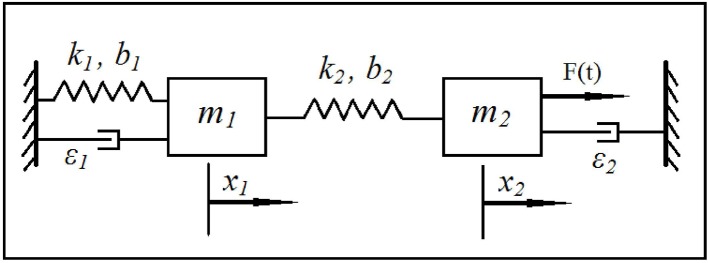
**Schematic of the stochastic coupled Duffing—van der Pol oscillators**.

We consider a phenomenological model of the EEG based on a coupled system of Duffing—van der Pol oscillators subject to white noise excitation, as shown in Figure 1. The equations for the model may be written as:

(3) $$\{\begin{array}{l} {\ddot{x}}_{1}+(k_{1}+k_{2})x_{1}-k_{2}x_{2}=-b_{1}x_{1}^{3}-b_{2}{\left(x_{1}-x_{2}\right)}^{3}\\ \;+\epsilon_{1}{\dot{x}}_{1}(1-x_{1}^{2}),\\ {\ddot{x}}_{2}-k_{2}x_{1}+k_{2}x_{2}=b_{2}{\left(x_{1}-x_{2}\right)}^{3}\\ \;+\epsilon_{2}{\dot{x}}_{2}(1-x_{2}^{2})+\mu \;dW, \end{array}$$

where *x*_*i*_, $\dot{x}$_*i*_, $\ddot{x}$_*i*_, *i* = 1, 2 are positions, velocities, and accelerations of the two oscillators, respectively. Parameters *k*_*i*_, *b*_*i*_, ϵ_*i*_, *i* = 1, 2 are, respectively, linear stiffness, cubic stiffness, and van der Pol damping coefficient of the two oscillators. Parameters *b*_*i*_s indicate the strength of the Duffing non-linearity resulting in multiple resonant frequencies while ϵ_*i*_s indicate the strength of van der Pol non-linearity and determine the extent of self-excitation and the shape of the resulting limit cycle. Parameter μ represents the intensity of white noise and *dW* is a Wiener process (Gardiner, 1985; Higham, 2001) representing the additive noise in the stochastic differential system. The input excitation to the system is provided through μ*dW*. The output may be selected as any combination of the positions and velocities to mimic an EEG signal. Note that, the Euler-Maruyama method (Higham, 2001) was selected to integrate the stochastic differential equations in Equation (3) since standard numerical integration methods are not applicable.

### 2.4. Optimization formulation

We have selected the velocity of the second oscillator as the system output approximating the EEG signal since it is directly impacted by the noise. A global optimization search method based on a multi-start algorithm (Ugray et al., 2007) was adopted to determine the oscillator model parameters that can produce the output matching various EEG signals. The optimization objective function was selected as the root mean squared of the errors in power spectrum of each selected brain frequency bands plus weighted values of the errors in absolute Shannon and sample entropies. Hence, the optimization goal is error minimization:

(4) $$\underset{p}{\min}J=\sqrt{\sum_{j\;=\;1}^{m}{\left(P_{E}{}_{j}-P_{O}{}_{j}\right)}^{2}}+w_{1}|S_{E}-S_{O}|+w_{2}|SP_{E}-SP_{O}|,$$

where *J* is the objective function, *p* = [*k*_1_, *k*_2_, *b*_1_, *b*_2_, ϵ_1_, ϵ_2_, μ] the decision variables, *P*_*E*_*j*__ and *P*_*O*_*j*__ the powers in the major brain frequency bands for the normalized EEG signal and the model output, respectively, *m* is number of frequency bands (*m* = 7), *S*_*E*_ and *S*_*O*_ the Shannon entropies of the EEG signal and the model output, respectively, *SP*_*E*_ and *SP*_*O*_ the sample entropies of the EEG signal and the model output, respectively, *w*_1_ and *w*_2_ are weighting factor for absolute Shannon and sample entropies, respectively, and | | represents absolute value. The weighting factors *w*_1_ and *w*_2_ are required to give equal importance to power spectrum and entropy characteristics of the signal. Note that the magnitude of the output signals are matched through normalization of both the model output and the EEG signal with respect to their standard deviations.

The objective function minimization is subject to equality constraints represented by the state (Equation 3) and inequality constraints represented by the decision variable lower and upper bounds:

(5) $$\begin{array}{l} 0<k_{i}\leq 1e4,\;0<b_{i}\leq \frac{1}{2}k_{i},\;0<\epsilon_{i}\leq \frac{1}{3}k_{i},\;\\ \;i=1,2,\;0\leq \mu \leq 2. \end{array}$$

The constraints for *b*_*i*_'s and ϵ_*i*_'s were imposed to avoid the chaotic regime (Li et al., 2006) and provide a periodic stochastic response. Noise intensity is also constrained to avoid a response dominated by random noise. The initial guesses for the global optimization search are randomly generated within the bounds defined in Equation (5).

The stochastic component was introduced as white noise, which was generated through a normally distributed random variable and applied to the model via Wiener process. A new random process was generated and applied to the model during integration of the equations, at each iteration of the optimization algorithm.

### 2.5. Statistical analysis

A key objective of the phenomenological modeling in this work is the ability to establish a correspondence between variations in model parameters and the variations in the data obtained from different physiological conditions. Hence, the parametric unpaired *t*-test and non-parametric Wilcoxon rank sum statistical testing methods were employed to determine the relative significance of the model parameters. Furthermore, Bonferroni correction was applied due to multiple comparisons problem and adequacy of sample sizes for statistical tests were established using power analysis.

### 2.6. Phase space reconstruction

In addition to matching Shannon and sample entropies of the model output and EEG signal through the optimization process, it is of interest to investigate matching other features such as the phase plot which plays a significant role in non-linear time series analysis (Kantz and Schreiber, 2004). It is known that any dynamic system can be completely recovered in the phase space, which maybe reconstructed from the measured time domain response of the system (Nie et al., 2013). While phase space consists of velocity and position variables for a mechanical system, in the case where just the time representation of a signal is available, a phase space reconstruction technique based on the method of delays is used (Kantz and Schreiber, 2004).

The main idea is that one does not need the derivatives to form a coordinate system in which to capture the structure of phase space, but instead one could directly use the lagged variables:

(6) $$x(n+T)=x(t_{0}+(n+T)\Delta \tau_{s}),$$

where *x*(*n*) is the *n*th sample of the time series, Δτ_*s*_ the time step, and *T* the delay integer to be determined. Then, a vector of (embedding) dimension *d* may be constructed using the time lags as:

(7) [x(n), x(n+T), x(n+2T), ⋯, x(n+(d−1)T)].

Time-delay embedding is probably one of the best systematic methods for converting scalar data to multidimensional phase space (Abarbanel et al., 1993; Burke and Paor, 2004; Nie et al., 2013). An appropriate and successful reconstruction depends on the choice of both time delay *T* and the embedding dimension *d* (Nie et al., 2013).

In this study, the appropriate value of the time lag was determined using the average mutual information method applied to each EEG recording block. The idea behind mutual information is to identify the amount of information that can be learned about a measurement at one time from a measurement taken at another time. Consider the time series *n*th sample *x*(*n*) and its value after time delay *T* with the associated probability distributions of *P*(*x*(*n*)) and *P*(*x*(*n* + *T*)), respectively. The average information which can be obtained about *x*(*n* + *T*) from *x*(*n*) is given by the mutual information of the two measurements (Abarbanel et al., 1993; Mizrach, 1996):

(8) $$I(x(n),x(n+T))=\log_{2}\left[\frac{P(x(n),x(n+T))}{P(x(n))P(x(n+T))}\right],$$

where *P*(*x*(*n*), *x*(*n* + *T*)) is the joint probability of the measurements *x*(*n*) and *x*(*n* + *T*) calculated using a binning-based method, in which 20 uniform intervals divided the range of the measurements equally. The average mutual information between measurements of any value *x*(*n*) and *x*(*n* + *T*) is the average over all possible measurements of *I*(*x*(*n*), *x*(*n* + *T*)) (Abarbanel et al., 1993):

(9) $$I(T)=\sum_{x(n),x(n+T)}P(x(n),x(n+T))I(x(n),x(n+T)).$$

If *T* is too small, the measurements *x*(*n*) and *x*(*n* + *T*) will have too much overlap. However, if *T* is too large, then *I*(*T*) will approach zero and nothing relates *x*(*n*) to *x*(*n* + *T*). It is suggested that the proper *T* can be chosen as the first minimum of *I*(*T*) which is not necessarily optimal but has been shown to work well (Abarbanel et al., 1993; Nie et al., 2013). If in a case, no minima exists for *I*(*T*), the choice of *T* = 1 or 2 has been suggested (Abarbanel et al., 1993).

After specifying the correct time delay *T*, an appropriate embedding dimension, *d*, should also be found for the phase space reconstruction. If *d* is too small, the trajectories will not be unique. On the other hand, too large a *d* will result in additional computational cost by requiring extra dimensions (Nie et al., 2013).

## 3. Results

The optimization algorithm was separately applied to determine the model parameters (decision variables) for each of the 60 selected EEG signals using the weighting factors *w*_1_ = *w*_2_ = 0.35. These weighting factors give equal importance to the entropy measures and power spectrum. We then categorized the resulting 60 set of model parameters into four groups based recording conditions and subject diagnosis: EC-CTL, EO-CTL, EC-AD, and EO-AD.

### 3.1. Healthy eyes-closed and eyes-open results

Initially, we studied the models derived for the EC and EO EEG signals of CTL subjects for validation purposes. The means and standard deviations of the optimal values of the model parameters for EC-CTL and EO-CTL EEG signals are listed in Table 1. The *p*-values from the two statistical tests and non-parametric method after Bonferroni corrections indicate that the differences between of all parameters of the two models are strongly statistically significant with the exception of noise intensity. Note that, μ is also found statistically significant using *t*-test but is slightly off when the non-parametric method is used.

**Table 1:** **Optimal parameters of the Duffing—van der Pol oscillator for EC and EO of CTL subjects (*N* = 40) and the *p*-values from unpaired *t*-test, Wilcoxon rank sum test, and Bonferonni correction**.

**Parameter**	**Eyes-Closed (EC)**	**Eyes-Open (EO)**	***t*-test**	**Wilcoxon**	**Bonferroni**
*k*_1_	7286.5 ± 192.4	2427.2 ± 448.91	1e-15	1e-8	1e-7
*k*_2_	4523.5 ± 282.3	499.92 ± 84.04	1e-15	1e-8	1e-7
*b*_1_	232.05 ± 18.3	95.61 ± 24.20	1e-15	1e-8	1e-7
*b*_2_	10.78 ± 2.3	103.36 ± 9.22	1e-15	1e-8	1e-7
ϵ_1_	33.60 ± 5.4	48.89 ± 9.49	1e-15	1e-8	1e-7
ϵ_2_	0.97 ± 0.19	28.75 ± 1.74	1e-15	1e-8	1e-7
μ	2.34 ± 0.47	1.82 ± 0.78	0.01	0.06	0.06

In order to ensure that adequate sample sizes are used, the minimum required difference between means of two groups of data for each parameter are computed. As expected due to very small *p*-values, the sample size for statistical testing is found to be sufficient with more than 99.9% power for all parameters except noise intensity μ, which was not found to be statistically significant using the non-parametric method.

**Figure 2:**
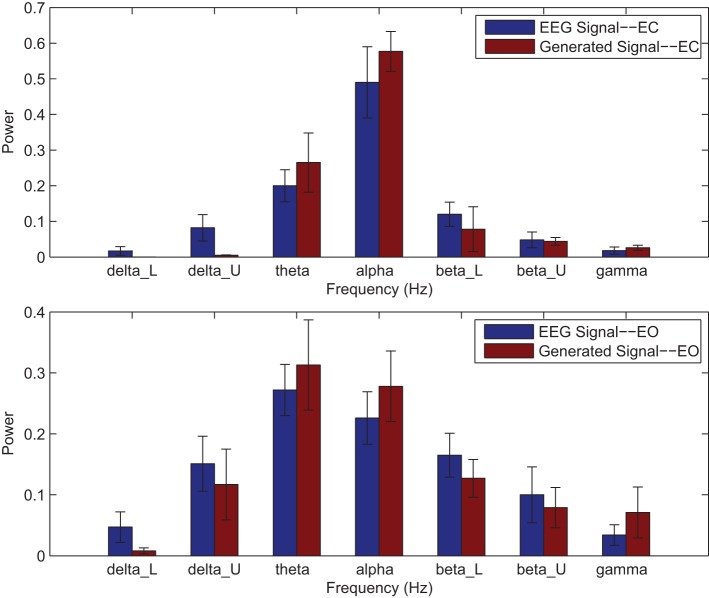
**Comparison of major brain frequency band mean powers of CTL EEG signals and optimal oscillator model output; EC (top), EO (bottom)**.

**Figure 3:**
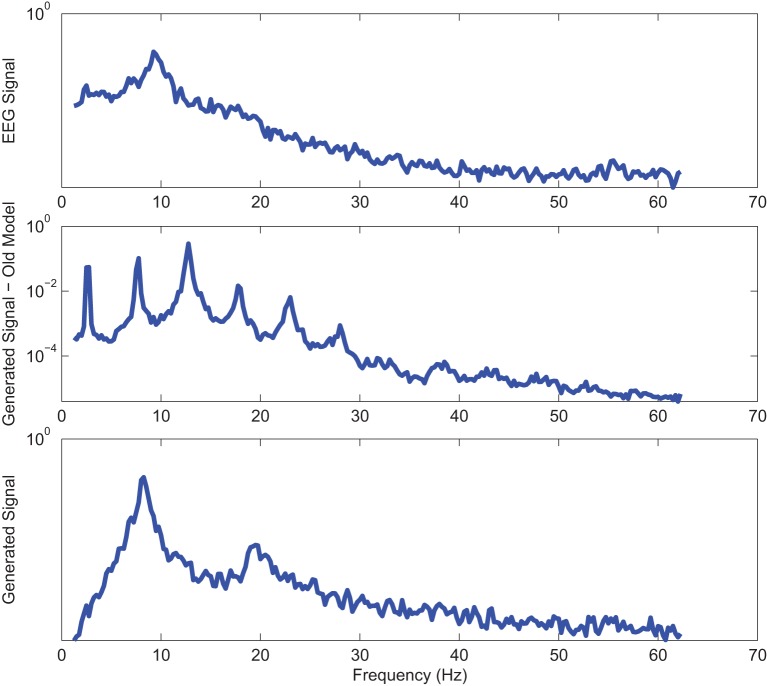
**Power spectrum of a sample CTL EC (top) EEG signal; (middle) output of stochastic oscillator model using Shannon entropy; (bottom) output of stochastic oscillator model using Shannon and sample entropies**.

**Figure 4:**
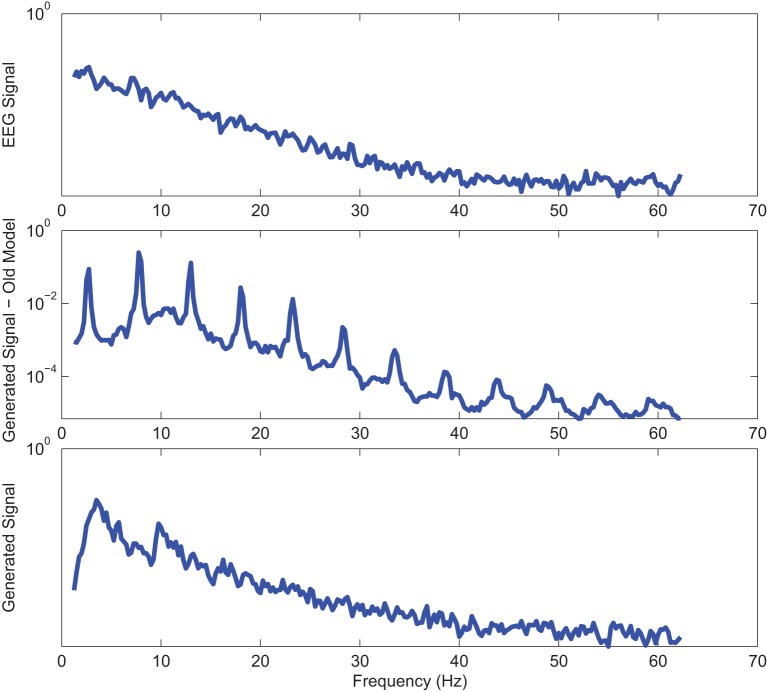
**Power spectrum of a sample CTL EO (top) EEG signal; (middle) output of stochastic oscillator model using Shannon entropy; (bottom) output of stochastic oscillator model using Shannon and sample entropies**.

Power spectrums of the optimal stochastic oscillator model output and EEG signals for the EC and EO cases of CTL subjects are presented in Figure 2 where θ, α, and β band powers show excellent agreement. The comparison revealed that, as expected, the optimal model is closely following the α-band dominance in the EC cases. While, in the EO cases, the optimal model follows a more flat frequency distribution from upper δ to lower β frequency bands. Furthermore, Shannon and SE values of the EEG signals and the model outputs for the EC and EO cases show close agreement. Shannon entropy values were 1.80 ± 0.08 and 1.92 ± 0.08 for EC EEG and model output, respectively, and 1.71 ± 0.11 and 1.57 ± 0.15 for EO EEG and model output, respectively. While, SE values were 1.04 ± 0.20 and 1.17 ± 0.22 for EC EEG and model output, respectively, and 0.97 ± 0.20 and 1.20 ± 0.18 for EO EEG and model output, respectively. These results show a significant improvement over our previous model where only Shannon entropy was used (Ghorbanian et al., 2015). The improvement is clearly observed in the the power spectra of sample EC and EO EEG signals and their corresponding optimal model outputs, respectively shown in Figures 3, 4. Both figures demonstrate more distributed spectra of the model outputs with similar noise complexities to the actual EEG signals when SE is added to the objective function; i.e., power spectra of the signals without matching of SE have very discrete peaks unlike the EEG.

**Figure 5:**
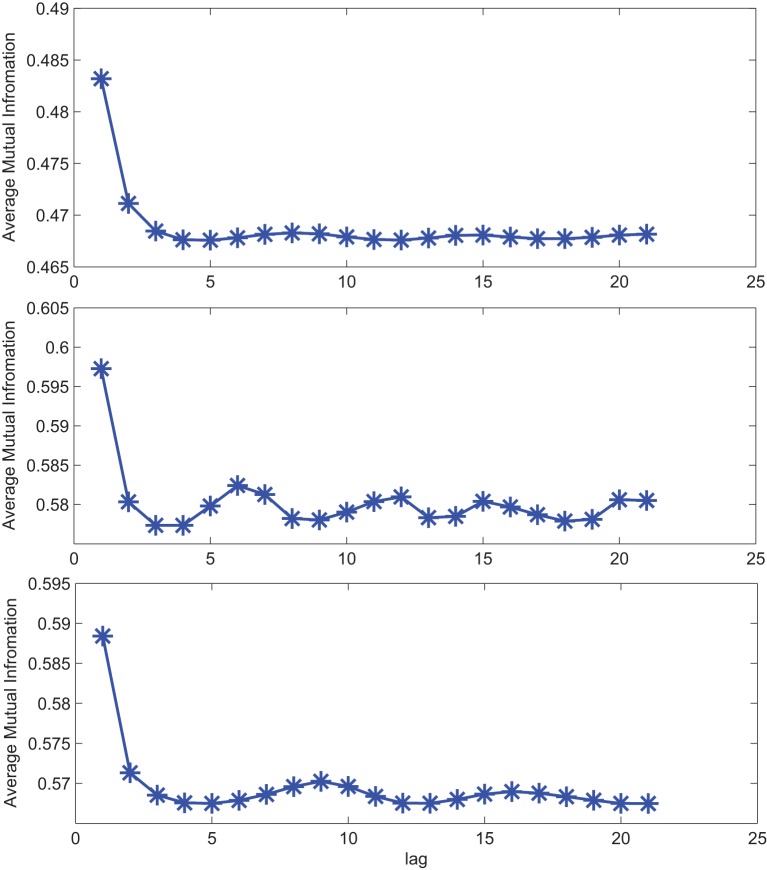
**Average mutual information for a sample CTL EC (top) EEG signal; (middle) output of stochastic oscillator model using Shannon entropy; (bottom) output of stochastic oscillator model using Shannon and sample entropies**.

**Figure 6:**
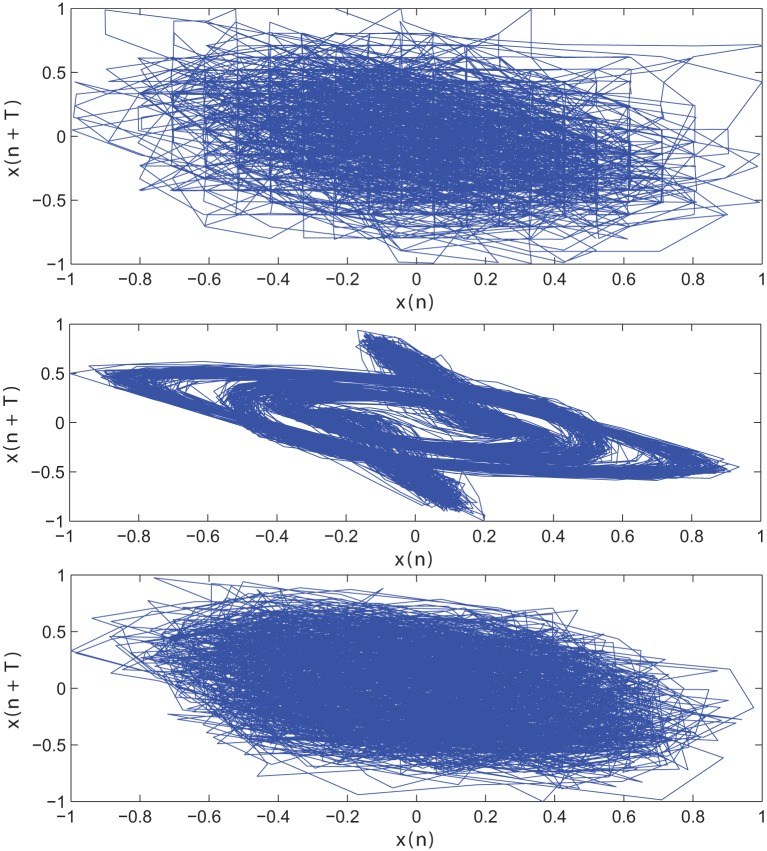
**Reconstructed phase plot of a sample CTL EC (top) EEG signal; (middle) output of stochastic oscillator model using Shannon entropy; (bottom) output of stochastic oscillator model using Shannon and sample entropies**.

The impact of SE to match signal complexity is further demonstrated through phase plot reconstruction of the time series. Average mutual information for a sample EC EEG signal and outputs of the optimal stochastic oscillator models are shown in Figure 5 as a function of lag time. The first minimum occurs at *T* = 5 lag samples for both the EEG signal and the optimal model derived with both Shannon and sample entropies while *T* = 3 for the output of the model derived solely based on Shannon entropy. The resulting reconstructed phase plots of the EC EEG signal and the outputs of the two optimal models are presented in Figure 6. Clearly, the reconstructed phase plots of the EEG and the output of the model derived using both Shannon and sample entropies, display similar behavior. While the output of the model derived using only Shannon entropy is qualitatively different form the EEG signal in terms of complexity and noise. Indeed this result provides further affirmation that the stochastic Duffing—van der Pol model yields an output that matches the actual EEG data in terms of non-linear characteristics observed in the phase space.

### 3.2. Alzheimer's disease vs. control results

Next, we studied the models derived for the EC and EO EEG signals of AD subjects. The mean and standard deviation of the optimal values of the model parameters for EC-AD and EO-AD EEG signals are listed in Table 2 along with the *p*-values from the two statistical tests and the non-parametric test after Bonferroni corrections indicating that the differences between only the first four parameters of the two models are statistically significant. Next, we separately compared the model parameters of EC and EO EEG signals of CTL subjects with those AD patients.

**Table 2:** **Optimal parameters of the Duffing—van der Pol oscillator model for EC and EO of AD subjects (*N* = 20) and the *p*-values from unpaired *t*-test, Wilcoxon rank sum test, and Bonferonni correction**.

**Parameter**	**Eyes-Closed (EC)**	**Eyes-Open (EO)**	***t*-test**	**Wilcoxon**	**Bonferroni**
*k*_1_	1742.1 ± 197.91	3139.9 ± 1040.9	0.0005	0.0025	0.009
*k*_2_	1270.8 ± 277.13	650.32 ± 175.76	1e-5	0.0005	0.002
*b*_1_	771.99 ± 126.81	101.1 ± 27.86	1e-12	0.0001	0.001
*b*_2_	1.91 ± 0.22	81.3 ± 9.76	1e-15	0.0001	0.001
ϵ_1_	63.7 ± 11.64	56.3 ± 5.75	0.0884	0.021	0.063
ϵ_2_	20.7 ± 5.64	19.12 ± 2.87	0.4234	0.879	0.95
μ	1.78 ± 0.8	1.74 ± 0.67	0.905	0.879	0.95

Table 3 lists the *p*-values from the two statistical testing methods and the non-parametric method after Bonferroni corrections comparing CTL vs. AD subjects under separate EC and EO conditions. The results indicate that the difference between all model parameters of CTL and AD subjects under EC condition are strongly statistically significant except for noise intensity. Again, μ is also found statistically significant using *t*-test but is slightly off when non-parametric method is used. The difference between the model parameters of CTL and AD subjects under EO condition are not, however, as strong, though they are still mostly statistically significant. In the EO case, parameter μ is not statistically significant using either method and *t*-test does not find *b*_1_ to be statistically significant either.

**Table 3:** **The *p*-values from unpaired *t*-test, Wilcoxon rank sum test, and Bonferonni correction for comparison of model parameters between AD (*N* = 20) and CTL (*N* = 40) subjects**.

**Parameter**	***t*-test (EC)**	**Wilcoxon (EC)**	**Bonf. (EC)**	***t*-test (EO)**	**Wilcoxon (EO)**	**Bonf. (EO)**
*k*_1_	1e-30	1e-5	4e-5	0.013	0.027	0.08
*k*_2_	1e-23	1e-5	3e-5	0.0034	0.0015	0.007
*b*_1_	1e-17	1e-5	5e-5	0.58	0.027	0.08
*b*_2_	1e-12	1e-5	6e-5	1e-6	1e-5	9e-5
ϵ_1_	1e-10	6e-5	1e-5	0.031	0.0018	0.007
ϵ_2_	1e-15	5e-5	7e-6	4e-12	1e-5	7e-5
μ	0.02	0.06	0.06	0.80	0.70	0.7

The power analysis results for 90%, 95%, 99%, and 99.9% for two statistical are listed in Tables 4, 5 for EC and EO cases, respectively. The actual difference between means are given within parentheses following each parameter. The results indicated that our sample size for statistical testing in EC case between AD and CTL subjects was sufficient for all parameters except μ with more than 99.9% power. However, in the EO case, only sample size for parameters *b*_2_ and ϵ_2_ has more than 99.9% confidence and *k*_2_ shows a 90% confidence. The sample size for the remaining parameters did not provide sufficient confidence.

**Table 4:** **Minimum required difference between model parameter mean values of EC AD vs. EC CTL for various desired powers of statistical tests**.

**Parameter**	**90%**	**95%**	**99%**	**99.9%**
Δ_*k*1_ (5544.3)	281.39	313.53	371.72	439.46
Δ_*k*2_ (3252.7)	406.65	453.09	537.18	635.08
Δ_*b*1_ (539.94)	106.44	118.60	140.61	166.24
Δ_*b*2_ (8.87)	2.82	3.14	3.72	4.40
Δ_*e*1_ (30.09)	11.54	12.86	15.25	18.03
Δ_*e*2_ (19.79)	4.64	5.17	6.13	7.25
Δ_μ_ (0.56)	0.87	0.97	1.15	1.36

**Table 5:** **Minimum required difference between model parameter mean values of EO AD vs. EO CTL for various desired powers of statistical tests**.

**Parameter**	**90%**	**95%**	**99%**	**99.9%**
Δ_*k*1_ (712.78)	1009.12	1124.36	1333.04	1575.97
Δ_*k*2_ (175.81)	175.81	195.89	232.25	274.57
Δ_*b*1_ (5.49)	36.86	41.07	48.69	57.56
Δ_*b*2_ (22.02)	13.61	15.17	17.98	21.26
Δ_*e*1_ (7.41)	12.27	13.68	16.22	19.17
Δ_*e*2_ (9.62)	3.14	3.50	4.15	4.91
Δ_μ_ (0.07)	1.08	1.21	1.43	1.70

**Figure 7:**
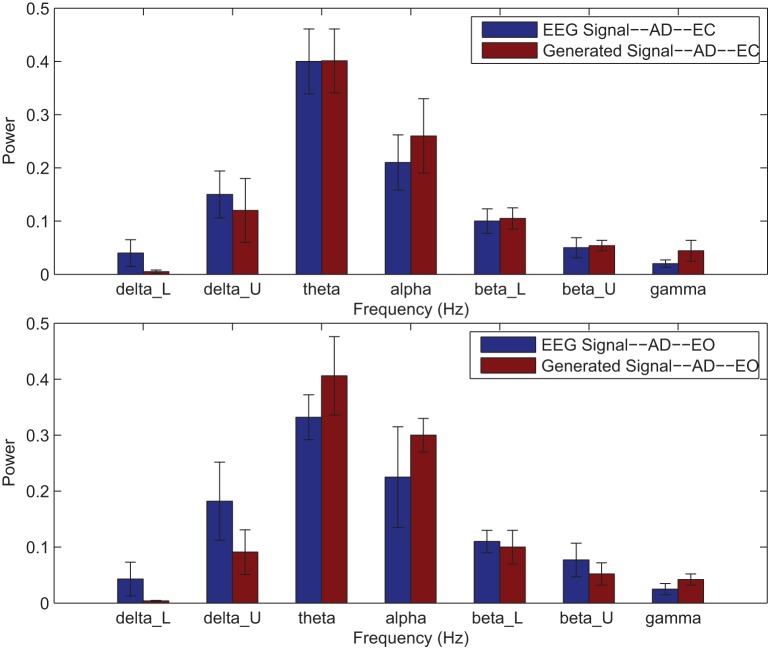
**Comparison of major brain frequency band mean powers of AD EEG signals and optimal oscillator model output; EC (top), EO (bottom)**.

Power spectrums of the optimal stochastic oscillator model output and EEG signals for the EC and EO cases of AD subjects are presented in Figure 7 where again θ, α, and β band powers show excellent agreement. The comparison revealed that the optimal model was closely and correctly slightly θ-band dominated in the EC cases for AD subjects (Ghorbanian et al., 2013). While, in the EO cases, the optimal model followed the more flat frequency distribution. Again, it should be noted that the higher error rates are related to those frequency bands with lower powers. Furthermore, Shannon and SE values of the EEG signals and the model outputs for the EC and EO cases show close agreement. Shannon entropy values were 1.78 ± 0.04 and 1.70 ± 0.10 for EC EEG and model output, respectively, and 1.63 ± 0.32 and 1.62 ± 0.27 for EO EEG and model output, respectively. While, SE values were 1.06 ± 0.19 and 1.17 ± 0.21 for EC EEG and model output, respectively, and 1.02 ± 0.39 and 1.29 ± 0.24 for EO EEG and model output, respectively.

**Figure 8:**
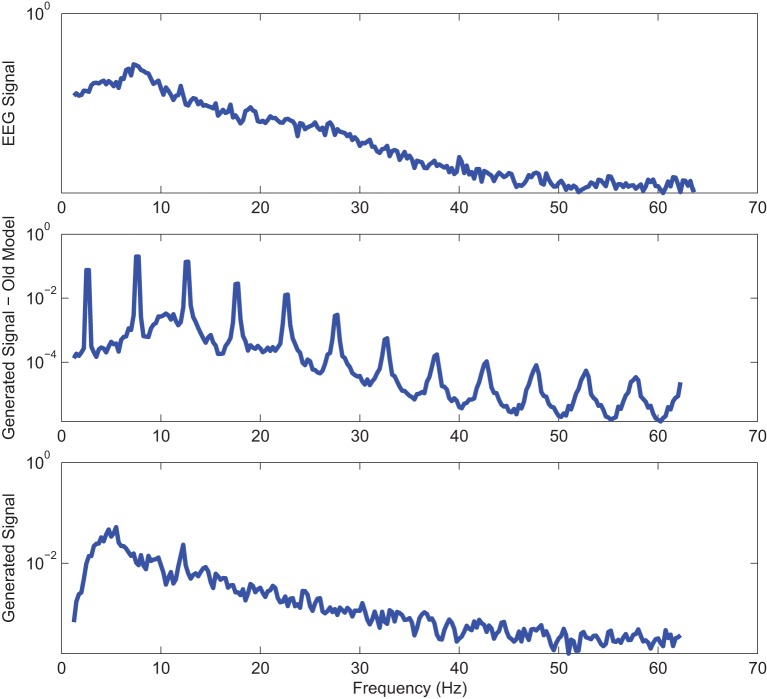
**Power spectrum of a sample AD EC (top) EEG signal; (middle) output of stochastic oscillator model using Shannon entropy; (bottom) output of stochastic oscillator model using Shannon and sample entropies**.

**Figure 9:**
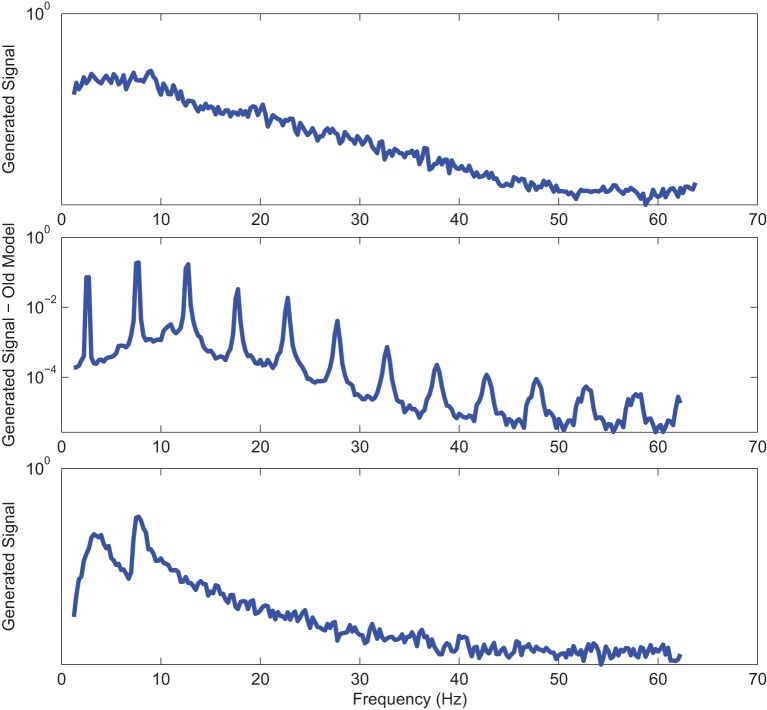
**Power spectrum of a sample AD EO (top) EEG signal; (middle) output of stochastic oscillator model using Shannon entropy; (bottom) output of stochastic oscillator model using Shannon and sample entropies**.

Power spectra of outputs of the optimal stochastic oscillator models and EEG signals for sample EC and EO cases of AD subjects are presented in Figures 8, 9. Again, it is clear that the addition of SE to the objective function results in output signals with power spectra patterns which are much more similar to the EEG signal in terms of distribution and noise complexity. As expected, the power spectrum plots demonstrated that the EC EEG signals from AD subjects were slightly θ band dominated unlike α band dominance of EC EEG recordings from CTL subjects.

## 4. Discussion

Power spectra of the optimal stochastic oscillator model output and EEG signals show excellent agreement in the brain's major frequency bands. The comparison revealed that the optimal model is closely following the α-band dominance in EC recordings for the control subjects. Furthermore, the model for EC recordings of AD patients closely followed θ-band power dominance indicating the slowing of the EEG signal for these patients. In the EO cases, the optimal model, as expected, followed a more flat frequency distribution from upper δ to lower β frequency bands for both AD and CTL subjects. Further evidence of robustness of the the models derived in this study is that the models derived for healthy subject EC and EO EEG signals in our earlier study (Ghorbanian et al., 2015) fall within the same distributions obtained for the CTL subjects in the clinical study.

Moreover, Shannon and SE values of the EEG signals and the model outputs for the EC and EO cases show close agreement for both CTL and AD subjects. However, the difference between the entropy values of the CTL subjects and AD patients were not statistically significant for neither the EEG signal nor the model output. This aspect needs to be further studied since EEG signals from AD patients may be expected to have lower complexity and thus lower entropy values.

The contributions of the article are as follows. Firstly, the objective function of the optimization scheme that yields model parameters based on comparison with actual EEG data in our previous work was extended to include both Shannon and sample entropies, with the latter being a measure of signal complexity. The procedure yielded model outputs that were in agreement with the actual EEG signals with respect to the frequency content (power spectra), information content (Shannon entropy) and complexity (sample entropy). It was shown that the addition of SE significantly enhances the performance of the optimal model in terms of both power spectrum and non-linear characteristics displayed through phase space reconstruction. The results demonstrate the feasibility of stochastic non-linear oscillator models which can be further studied for greater insight into EEG signal dynamic characteristics.

Secondly, the model parameter differences for EC and EO EEG recordings were statistically significant leading to qualitatively and quantitatively distinct realizations of the underlying models for the cases considered. This is a key result of the work since it verifies that distinct models represent the EEG signals recorded under different brain states. Potentially, this could lead to unique models for different brain disorders and injuries.

Thirdly, the study provided unique models for EC and EO EEG recordings from AD patients. The results showed that almost all of the model parameters were statistically significant for the EC and EO cases when comparing the AD and CTL subjects. Moreover, the power spectrum plots showed a good match between the generated signal from the stochastic model and the actual EEG signal from AD patients. However, the results for the EC case of AD were more accurate and reasonable than the results of EO cases mainly due to the ability of the optimization scheme to provide a better match in EC cases. The important conclusion here is that unique stochastic non-linear oscillator models can be developed to represent EEG signals from patients with a brain disorder.

Of particular interest is the potential connection between our model and the neural mass models studied in the literature. For instance, characterization of functional connectivity between remote cortical areas has been studied using neural mass models (David and Friston, 2003; David et al., 2004). These and other efforts (Sotero et al., 2007) represent intriguing attempts to capture actual neural dynamics using coupled oscillator models and suggest that, after all, models such as the one discussed in this article may be of broader scope than being purely phenomenological. Extrapolating further, it would then be of immense interest to understand the manifestation of phenomena such as synchronization (Mirollo and Strogatz, 1990) within the framework of our model and the implications for EEG characterization.

## 5. Conclusions

In this article, we presented results that further develop our recent work on modeling the EEG signal as the response of a stochastic, coupled Duffing—van der Pol system of two oscillators. The results presented verify that unique and statistically significant stochastic Duffing—van der Pol oscillator models represent EEG recorded from AD patients vs. health controls. Overall, the results presented in this article further affirm the efficacy of a stochastic Duffing—van der Pol oscillator network model in capturing the key characteristics of actual EEG data under different brain states as well as brain conditions in terms of healthy controls vs. patients with a brain disorder. The validation provided by the results certainly motivates further research toward improving the analytical model and testing it against larger data sets. Furthermore, the results suggest that the modeling approach could potentially help develop novel diagnostic and interventional tools for neurological diseases and disorders.

### Conflict of interest statement

The authors declare that the research was conducted in the absence of any commercial or financial relationships that could be construed as a potential conflict of interest.
